# The Impact of Dry Yeast Rehydrated in Different Plasma Treated Waters (PTWs) on Fermentation Process and Quality of Beer

**DOI:** 10.3390/foods11091316

**Published:** 2022-04-30

**Authors:** Aneta Pater, Paweł Satora, Marek Zdaniewicz, Paweł Sroka

**Affiliations:** Department of Fermentation Technology and Microbiology, Faculty of Food Technology, University of Agriculture, Balicka Street 122, 30-149 Kraków, Poland; pawel.satora@urk.edu.pl (P.S.); m.zdaniewicz@urk.edu.pl (M.Z.); pawel.sroka@urk.edu.pl (P.S.)

**Keywords:** dry yeast, ale yeast, lager yeast, fermentation, beer, plasma treated water

## Abstract

Yeast plays a key role in the production of alcoholic beverages. Effective fermentation requires appropriate conditions to ensure the production of high-quality beer. The paper discusses the effect of dry brewing yeast (*Saccharomyces cerevisiae* and *Saccharomyces pastorianus*) after rehydration with water exposed to low-temperature, low-pressure glow plasma (PTW) in the atmosphere of air (PTWAir) and nitrogen (PTWN) in the course of the fermentation process, the formation of volatile compounds and other quality parameters of the finished beer. The obtained results show that the lager yeast strain initiated the process of fermentation faster after rehydration in the presence of PTWAir compared to all of the other treatments. It was observed that PTWAir significantly changed the composition of volatile compounds in the finished beer, especially by increasing the number of terpenes, which are compounds that positively shape the aroma of beer. In the case of PTWN samples, lower alcohol content, real extract, apparent extract and amount of biomass were observed in all analyzed strains.

## 1. Introduction

Beer is one of the most widely used alcoholic beverages in the world [1]. The taste and aroma of beer depend on the type of used malt and hops, the course of the fermentation process and beer storage conditions [2]. The main compounds that influence the aroma of alcoholic beverages are esters, higher alcohols, terpenes, and acids [3]. During the fermentation process, yeast contributes to the formation of some volatile compounds [4]. That is why the yeast strain as well as its viability and vitality are very important for obtaining high-quality beer. In industrial breweries, it is possible to ensure the appropriate quality of yeast biomass thanks to the propagation stations [5]. In the case of craft breweries, the number of yeast propagation stations is increasing [6]. Breweries without propagation stations use liquid yeast from commercial supplier or dried yeast for fermentation. The advantages of dry yeast compared to fresh yeast include ease (no need to cultivate) and convenience of use (storage), quality (no contamination) and homogeneity [7]. However, during the processes of yeast dehydration and subsequent rehydration yeast cells may be exposed to a series of physiological stresses. An increase in the osmotic pressure may have a determinantal effect on the membranes, leading to structure changes [8]. Yeast cells survive complete dehydration and quickly resume metabolic activity upon re-contact with water [9]. Yeast tolerates a lack of water due to the ability to synthesize large amounts of trehalose [10]. Therefore, scientists have made attempts to improve the physicochemical qualities of the obtained yeast biomass after rehydration. In their research, Leslie et al. [11] conditioned yeast cells with the use of trehalose. Beker and colleagues [12] investigated the effect of vacuum-dried yeast storage conditions under nitrogen on their rehydration. Research conducted by scientists has shown that the addition of trehalose and an increased temperature of yeast irrigation prevents the negative effects of reducing their viability. All of the above-mentioned studies ultimately resulted in an increase in the viability of yeast cells after rehydration. Therefore, it is important to use an appropriate medium and rehydration conditions, which may contribute to the improvement of the yeast condition after its earlier drying process. The temperature of the rehydration medium, which is important during rehydration, should be higher than the phase transition temperature of the cell membrane [7]. Another important factor is the time which should not exceed 30 min [13].

The presented article is a continuation of research on the possibility of using plasma treated water (PTW) in the brewing industry [14,15]. A device developed in 2009 by Elkin et al. [16] has been used for the production of PTWs. The first studies which explored the possibility of using this water appeared in the fields of cosmetology and medicine [17]. More research continues to appear in animal husbandry and the cultivation of plants [18,19,20]. Szymanowicz et al. [19] investigated the effect of plasma-treated water as a storage solvent for commercial boar semen; they showed that the boar semen activity after stored in PTW was higher from the 5th to the 13th day, compared to the control sample. Ciesielska et al. [21] investigated the influence of PTW on the cultivation of cress. After watering the plants with this water, the yield of cress, protein and fat content was increased. The studies carried out so far show that the properties of PTWs depend on the time of water exposure to the plasma and the type of water used for this purpose [22]. When plasma is operated on water, depending on the gas atmosphere, active forms of reactive oxygen (ROS) or nitrogen species (RNS) are formed. Scientific research shows that the resulting components, ROS and RNS, may have antimicrobial effects depending on their amount, which depends on the duration of plasma exposure [23]. Their small amount can affect the cells by activating the cellular response to stress factors, especially oxidative stress [24]. Oxidative stress resistance is a measure of a cell’s resistance to a variety of environmental factors, including osmotic stress, ethanol, temperature, and more. Cells exposed to oxidative stress are better prepared for adverse conditions and adapt to them more easily [20].

This article examines how yeast rehydrated with PTWs [20] will affect the fermenta-tion process and the quality of the finished beer. Dry yeasts of top and bottom fermenta-tion strains (Saccharomyces cerevisiae Safbrew US-05, T58, S33 and Saccharomyces pastorianus Saflager W34/70) were rehydrated in the presence of plasma water (PTWAir, PTWN). To determine whether yeast rehydrated with PTWs could improve the fermentation process, the formation of volatile compounds and other quality parameters of the finished beer were analyzed. The results were compared with the control sample (yeast rehydrated with water without plasma—CW).

## 2. Materials and Methods

### 2.1. Materials

The biological material for the analysis were the strains Saccharomyces cerevisiae (Safbrew US-05, T58, S33) and Saccharomyces pastorianus (Saflager W34/70) (Lesaffre Fermentis, Warsaw, Poland) [15].

For plasma treatment (PTWAir, PTWN) commercially available Żywiec Zdrój (Żywiec Zdrój, Danone, Cięcina, Poland) spring water was used. The control water (CW) was the spring water without plasma treatment.

For malt wort production, commercially available pilsner malt was used (Viking Malt), and the Polish hop variety Marynka (7.5% alpha acids) (Polish Hops, Karczmiska Pierwsze, Poland).

### 2.2. Methods

The device presented and described in the previous scientific article [14] was used for the production of plasma treated water in the research.

#### 2.2.1. Plasma Treated Water under Air Atmosphere (PTWAir)

Spring water was used in the plasma process (Żywiec Zdrój, Danone, Cięcina, Poland). A total of 1500 mL of this water was placed in an open 2000 mL Pyrex glass bottles. Then, this bottle was placed in a reactor chamber close to a lamp generating plasma for 30 min. The lamp generated plasma at 38 °C under 5 × 10^−3^ mbar, 600 V, 50 mA and 10 kHz. The chamber with the water sample remained at normal pressure [14]. The water was used immediately after plasma was performed.

#### 2.2.2. Plasma Treated Water under Nitrogen (PTWN)

Spring water (1500 mL) was placed in an open 200 mL Pyrex glass bottles and N_2_ was bubbled through the water for 15 min. N_2_ was deoxygenated by passing it through an absorber filled with alkaline solution of resorcinol. After placing the bottles in the reactor, its chamber and free space over the liquid were additionally filled with deoxygenated N_2_. Treatment conditions with LPGP and storage of the product are described in PTWAir. 

#### 2.2.3. Beer production

##### Milling, Mashing and Lautering

All-malt mashes were prepared in Mash Bath R12 (1-CUBE, Czech Republic) according to the method EBC 4.5.1. [25]. To obtain 12 °P extract, increased the malt dose from 50 to 75 g. The malt was milled in a disc mill, weighed into tarred mash containers, then placed in a water-heated apparatus at 45 °C. Agitators were set up and the “Congress program” was selected. Next, 200 mL of distilled water at 45 °C was poured in portions into the containers. The apparatus was held at 45 °C for 30 min. Then, the temperature was raised at a rate of 1 °C/min until it reached 70 °C, with constant stirring of samples. When the apparatus reached 70 °C, 100 mL distilled water was warmed to the same temperature and added to the cups, and then the set temperature was maintained for 1 h. Next, the containers were cooled to 20 °C and filled with distilled water up to a mass of 450.0 g and filtered through a paper filter (MN614). To ensure high clarity, the first portions of the filtrate were recirculated.

##### Boiling

The obtained wort was combined and boiled. At the beginning of the boiling process in heating mantle, Marynka variety hops (7.5% alpha acids) at an amount of 2.62 g was added to the 3270 mL of wort. After 60 min of boiling, the hot tub was removed from the wort using Grade 802 Whatman filter paper. Subsequently, the samples were cooled to 20 °C. Before inoculation, the key quality parameters of the wort were analyzed (Table 1).

##### Rehydration of Dry Yeast

Dry yeast (1 g) was suspended in 10 mL of water (PTWAir, PTWN, CW) at 25 °C. The yeast was left to rest for 15 min, and after this time, gently stirred for 30 min as recommended by the manufacturer.

##### Fermentation Trials

Prepared hopped wort was inoculated with yeast cell *Sc* (US-05, T58, S33) to obtain live yeast cell suspensions 1 × 10^6^ cells/mL and *Sp* W34/70—2 × 10^6^ cells/mL. The wort extract was also taken into account while the appropriate number of yeasts was added. The number of cells in 1 mL of suspension was evaluated using the Thoma chamber (in tripticale). All of the samples (300 mL) were fermented in rubber-stoppered Erlenmeyer flasks with fermentation tubes, at 20 °C for 8 days in the case of ale yeast, and 10 °C for 13 days in the case of lager yeast. Fermentation was carried out until weight loss was observed, in case it was no longer observed the process was completed earlier (Q-CELL 240 thermostatic chamber, Wilkowice, Poland).

After fermentation, yeast suspension was separated from the beer by centrifugation (Centrifuge MPW-365, Warsaw, Poland).

### 2.3. Analytical Determinations

#### 2.3.1. The International Bitterness Unit (IBU)

This analyzes was measured with the use of isooctane extraction of iso-*α*-acids from acidified samples. After extraction, the absorbance analysis was conducted (Beckman DU-650 UV-Vis, East Lyme, CT, USA) with the use of a 275 nm wavelength according to Analytica EBC method 8.8 [26].

#### 2.3.2. Ethanol, Real and Apparent Extract

These analyzes were measured with an automatic beer analyzer (Alcolyzer, Anton Paar DMA 4500+, Warsaw, Poland). The samples were degassed by mixing beer with diatomaceous earth and filtered through filter paper to obtain 50 mL of filtrate. The filtrate was degassed for 20 min (universal shaker, 150 rpm), brought to 20 °C and filtered again.

#### 2.3.3. Ethanol Yield

The predicted values for the ethanol yield were calculated according to Charles et al. [27] with the formula:

(1) $$\mathrm{Ethanol}\;\mathrm{yield}\;[g/g]=\frac{\mathrm{Alcohol}\;\left(\%\frac{w}{w}\right)}{\left(\mathrm{Real}\;\mathrm{wort}\;\mathrm{extract}\;-\;\mathrm{Real}\;\mathrm{beer}\;\mathrm{extract}\right)}$$

#### 2.3.4. The pH of the Obtained Wort and Beer

The pH of the wort and beers was measured using the Mettler Toledo FiveGO pH meter (Warsaw, Poland).

#### 2.3.5. The Colour of the Obtained Wort and Beer

The filtered wort and beers were measured spectrophotometrically (Beckman DU-650 UV-Vis) at a wavelength of 430 nm according to Analytica EBC [28].

#### 2.3.6. Free Amino Nitrogen (FAN)

The FAN was measured using ninhydrin-based methods with the use of the absorbance measurement at 570 nm (Beckman DU-650 UV-Vis) according to the Analytica EBC [29] method 8.10_Free Amino Nitrogen in wort by Spectrophotometry (IM).

#### 2.3.7. Volatile Compound Analysis SPME-GC/FID

In order to determine the headspace volatile compounds, 1 g of NaCl and a 2 mL sample of beer were placed into a 10 mL vial. Next, an internal standard solution was added (0.57 mg/L 4-methyl-2-pentanol, 0.2 mg/L anethol and 1.48 mg/L of ethyl nonanoate, Sigma-Aldrich, Saint Louis, MO, USA). The SPME device (Supelco Inc., Bellefonte, PA, USA) coated with PDMS (100 μm) fiber was first conditioned by inserting it into the gas chromatograph injector port at 250 °C for 1 h. For sampling, the fiber was inserted into the headspace under stirring (300 rpm) for 40 min at 40 °C. Subsequently, the SPME device was introduced into the injector port of the Hewlett Packard 5890 Series II chromatograph system, and kept in the inlet for 3 min. The tested components were separated on a Rxi^®^-1 ms capillary column (Crossbond 100% dimethyl polysiloxane, 30 m × 0.53 mm × 0.5 μm). The detector temperature was 250 °C, and the column was heated using the following program: 35 °C for four min at an increment of 5 °C/min to 110 °C and then an increment of 20 °C/min to 230 °C, finally maintaining a constant temperature of 4 °C. The carrier gas was helium at a 1.0 mL/min constant flow.

The qualitative and quantitative identification of volatile substances (ethyl acetate, ethyl hexanoate, ethyl octanoate, 2-phenylethyl acetate, ethyl decanoate, ethyl dodecanoate, 2-methyl-1-propanol, 2-methyl-1-butanol, 2,3-butanediol, 1-hexanol, 2-phenylethanol, 1-nonanol, β-myrcen, linalool, geraniol, β-damascenone, humulene, β-farnesene and nerolidol; Sigma-Aldrich) were based on a comparison of retention times and peak surface area reads from samples and standard chromatograms. This method was validated based on the method described by Antalick et al., 2010 [30].

#### 2.3.8. Organic Acid Analysis

High-performance liquid chromatography was applied for the analysis of the organic acids. An HPLC analysis was carried out on a Perkin-Elmer (USA) FLEXAR chromatograph (Waltham, Massachusetts, USA) with a UV-Vis detector. Malic, succinic, lactic, citric, and acetic acids (Sigma-Aldrich) were determined using the Rezex ROA-Organic Acid Aminex HPX-87H (300 mm, 18 cm × 7.8 mm). Samples were isocratically eluted at 40 °C with a mobile phase (0.005 MH2SO4) at a flow rate of 0.4 mL/min. Quantitative determinations were made with the use of standard curves prepared with the appropriate standards: malic, succinic, lactic, citric, and acetic acids [31].

#### 2.3.9. Content and Profile of Sugars

An analysis of the sugar profile was conducted with the Shimadzu (Kyoto, Japan) NEXERA XR apparatus with an RF-20A refractometric detector. The separation was conducted with an Asahipak NH2P-50 4.6 × 250 mm Shodex column (Showa Denko Europe, Munich, Germany) thermostated at 30 °C. The mobile phase consisted of an acetonitrile aqueous solution (70%), and the isocratic elution program (0.8 mL/min) lasted for 16 min. Quantitative determinations were made with the use of standard curves prepared with the appropriate standards: glucose, maltose, and glycerol [31].

#### 2.3.10. Determination of Metal Ions

Magnesium, calcium, and zinc ions were analyzed in wort and beers samples with VARIAN 20FS spectrometer (AA240FS, Ontario, Canada) based on atomic absorption spectrometry with flame atomisation (air/acetylene). The instrument used an SPIS-20 automatic sample proportioning system. The absorbance of Mg^2+^ was determined at 202.6 nm, Ca^2+^ at 422.7 nm, and Zn^2+^ at 213.9 nm.

### 2.4. Statistical Analyses

The results are presented as the mean of four independent replicate experiments. The Shapiro-Wilk test was applied to assess the normality of the data distribution. The data were analyzed using a two-way analysis of variance (ANOVA) and one-way analysis of variance (ANOVA). The significance in the difference for each parameter was analyzed separately using Tukey’s post hoc test (Statistica v.10, StatSoft Inc., Krakow, Poland) and heat-map test (MS Excel).

## 3. Results

### 3.1. Fermentation Dynamics

**Figure 1:**
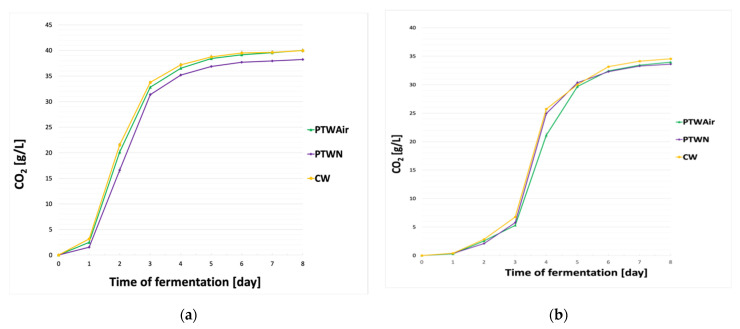

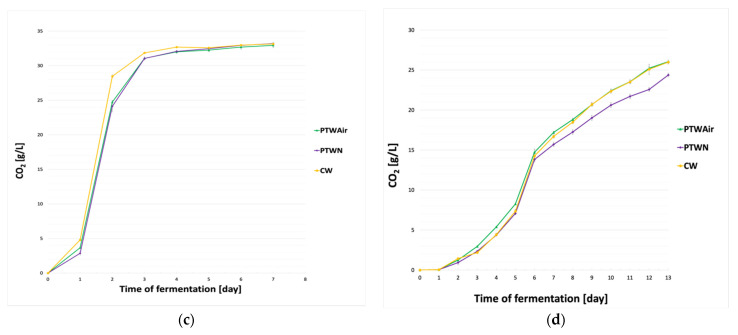
Fermentation kinetics of (**a**) *S. cerevisiae* US-05, (**b**) *S. cerevisiae* T58, (**c**) *S. cerevisiae* S33, (**d**) *S. pastorianus* W34/70 after rehydration with PTWAir, PTWN and CW; *n* = 4.

The kinetics of fermentation was monitored by measuring the amount of released carbon dioxide (g/L) over the consecutive days of the process. From the moment of inoculation of the wort, in the case of the *Sc* US-05 yeast strain (Figure 1a), significant differences were observed on the first day of fermentation. The speed of fermentation with the yeast rehydrated with PTWN was significantly lower compared to the yeast rehydrated with control water and PTWAir. This suggests that the extent of sugars consumption by yeast was reduced. However, no significant differences were found between the yeast rehydrated with plasma treated waters. This trend continued until 3rd day of fermentation. From the 4th day, we noticed that there were statistically significant differences between the yeast rehydrated with PTWAir and yeast rehydrated with PTWN. In the case of the yeast strain *Sc* T58 (Figure 1b), it was observed that the differences in fermentation appeared only on the second day of the process. From the 2nd to 3rd day, yeast samples rehydrated with control water were characterized by highest fermentation rate. In the case of yeast rehydrated with plasma treated waters samples were fermented at a similar way from the 4th day, the yeast rehydrated with PTWN began to show similar weight losses as the yeast rehydrated with control water. The yeast rehydrated with PTWAir fermented the weakest in this case. On day 6, the yeast rehydrated with PTWN reduced their weight loss significantly slower than for control water samples, and similar to the yeast rehydrated with PTWAir samples. From the very beginning of fermentation to the last day of its duration, the yeast samples rehydrated with CW showed the highest fermentation rate.

During the fermentation of the wort with yeast strain *Sc* S33 (shown in Figure 1c), the largest differences were observed between the second and third day of the entire process, where the yeast rehydrated with plasma treated waters significantly differed from the yeast rehydrated with CW. On the remaining days, no statistically significant differences were observed in the course of the fermentation process between the samples.

Fermentation with the lager yeast strain *Sp* W34/70 (Figure 1d) lasted for 13 days. It was observed that the yeast rehydrated with PTWAir fermented more efficiently from the 1st to the 5th day of the process. Differences were also observed from the 6th day of the fermentation process. The yeast rehydrated with PTWN showed significantly smaller weight losses compared to yeast rehydrated with PTWAir and CW.

### 3.2. Ethanol, Real Extract, Apparent Extract and Apparent Degree of Fermentation

The alcohol content in the analyzed beers fermented by *S. cerevisiae* strains ranged from 4.30% to 5.35% (*v*/*v*), while by *S. pastorianus*—from 4.51% to 4.79% (*v*/*v*) (Table 2). In the obtained results, we noticed that different medium used for yeast rehydration signifi-cantly contributed the alcohol content in all of the analyzed beers. The sample of *Sc* US-05 strain was characterized by the highest alcohol content, compared to other strains, respec-tively, 5.26% to 5.35% *v*/*v*. The lowest alcohol amount was found in the samples ferment-ed by the yeast strain *Sc* S33 (4.30% to 4.48% *v*/*v*). A similar trend was observed in the case of the *Sc* T58 and *Sp* W34/70 strains. In both cases, rehydration in the presence of PTWN contributed to a significant reduction in alcohol content when compared to CW.

The lowest content of real extract was found in beers fermented with yeast strain *Sc* US-05 (around 4.1% *w*/*w*). Only in the case of this yeast strain, the use of PTWN for rehydration resulted in a reduction of the real extract after fermentation, more than in the case of using other waters for rehydration (Table 2). Generally, samples fermented with yeast rehydrated in PTWAir were characterized by increased level of real extract. Ethanol yield results showed that PTWs contributed to obtaining a lower content of this parameter in beers fermented with *Sc* US-05 and *Sc* T58. The beer with lager yeast strain rehydrated with PTWN had the lowest ethanol yield.

### 3.3. Amount of Biomass

After completion of the fermentation process, the yeast biomass was separated from the beer by centrifugation. The highest amount of biomass was produced by the yeast strain *Sc* US-05 and *Sc* S33 (Table 3), the lowest by the lager strain (0.76 to 2.21 g of dry mass/L). Yeast rehydrated in PTWN produced less biomass during fermentation (Table 3). In the case of the lager strain *Sp* W34/70, the amount of biomass was decreased by 66% when yeast were rehydrated in the presence of PTWN compared to the control water.

### 3.4. Colour, pH and FAN

The rehydration of yeast in the presence of plasma treated waters significantly influenced the colour of the obtained ale beers. In ale beers, rehydration in PTWs decreased intensity of the colour, while in the lager beers increased. The lowest value of this parameter was detected in the samples obtained with yeast *Sc* US-05 rehydrated in the presence of PTWAir (5.62 EBC units). In the case of the yeast strain *Sc* T58, rehydration in the presence of PTWAir and PTWN caused a reduction in the colour of the beer (7.87 EBC and 7.22 EBC, respectively) compared to the samples obtained with the rehydrated culture in the control water (8.12 EBC). In the case of the lager beers, the samples rehydrated with PTWAir showed the the most intense colour compared to the other variants (Table 3). 

The pH value of the wort before starting the fermentation process was 5.75 (Table 2). After completion of the fermentation process, we noticed that the water used for the rehydration of the yeast did not significantly influence the pH of beers (Table 3). A significant statistical difference was noticed in the case of the *Sc* US-05 yeast strain, which lowered the pH value of the beers, compared to the other strains.

The wort used for the research with the 11.9 °P extract contained 191 mg/L of free amino nitrogen (FAN). After the end of the fermentation process, the content of free amino compounds in the obtained beers was also analyzed. We noticed that plasma treated water used for rehydration of dry brewer’s yeast did not significantly influence this parameter. However, differences between the tested yeast strains were found. The lowest FAN content was observed in beers produced with the *Sc* US-05 and *Sc* T58 yeast strains (Table 3). The highest FAN content was observed in the samples fermented with *Sp* W34/70 lager yeast (131 to 142 mg/L). This provides evidence that the ale yeast used more nitrogen components for their growth.

### 3.5. Ion Content

The wort, as well as obtained beers, were also analyzed for the content of three ions (magnesium, calcium and zinc) important for brewing. The wort contained 93.7 mg/L Mg^2+^, 21.6 mg/L Ca^2+^ and 0.91 mg/L Zn^2+^, respectively. Water used for yeast rehydration did not significantly affect the utilization of all of the ions during the fermentation process, however, the yeast strains analyzed differed in their demand for these ions (Table 3).

### 3.6. Sugars and Organic Acids

Before starting the fermentation process, the wort contained 37.13 g/L of maltose, 7.74 g/L of glucose and 8.92 g/L of fructose (Table 1), respectively. After fermentation, it was observed that the rehydration of yeast in the presence of plasma treated water significantly influenced the content of maltose and fructose in the analyzed beer samples (Table 3). Overall, the use of PTWAir for rehydration caused the yeast to consume more maltose during fermentation, and the PTWN reduced the amount of maltose used compared to control sample. This tendency is best seen in the case of *Sp* W34/70 yeast. In beers obtained by the aforementioned strain of yeast, the most unfermented maltose remained, while the least remained in the samples fermented with T58 and US-05 ale yeast.

In the case of fructose, the opposite trend was observed, and a negative Pearson correlation coefficient was found between the content of these two sugars in beers. The highest content of fructose was detected in beers obtained with the *Sc* T58 strain (Table 3). Plasma treated water used for rehydration also significantly contributed to the changes in the content of this sugar in the final product. For almost all of the yeast strains (except *Sc* T58) their rehydration in the presence of PTWAir contributed to the highest fructose content in the analyzed samples, while CW showed the lowest residual fructose.

Noteworthy are the results of the content of glycerol in beer (Table 3). Glycerol was detected at concentrations from 4.41 to 8.93 g/L. The glycerol content in lager beers (8.03–8.93 g/L) was much higher than in ale (up to 6.68 g/L). A significant effect of plasma treated water for yeast rehydration on the glycerol level in produced beer was also observed. In the case of the *Sc* US-05 and *Sc* S33 yeast strains, rehydration in the presence of PTWN significantly contributed to the reduction in glycerol content in the beers, compared to the samples obtained using yeast rehydrated with CW. On the other hand, beers fermented with the *Sc* S33 and *Sp* W34/70 yeast strains rehydrated in the presence of PTWAir contained the highest glycerol concentration compared to the others samples (Table 3).

Acetic, lactic, malic, citric, and succinic acids were present in the produced beers. Their level depended on the strain applied for fermentation and not on the water used for rehydration. Beers produced with the lager yeast strain contained more acetic acid and almost non-detectable amounts of other analyzed organic acids. Ale beers were characterized by higher levels of malic, citric and succinic acids; however, significant differences were found between the *S. cerevisiae* strains.

### 3.7. Volatile Compounds

The aroma composition of all of the analyzed beers was strongly modified by water used for yeast rehydration and their strain. Among analyzed components in the finished beers, esters and higher alcohols were present in the highest concentrations. The following esters dominated in the fermented samples: ethyl acetate, 2-phenylethyl acetate and ethyl decanoate (Table 4). In the case of higher alcohols, 2-methyl-1-butanol, 2-phenylethanol and 1-hexanol were found in highest concentrations (Table 5). The rehydration of yeast with PTWAir reduced in beers the concentration of some esters such as 2-phenylethyl acetate (samples after fermentation with *Sc* US-05 strain). On the other hand, beers fermented with *Sc* US-05 yeast after rehydration with PTWN had a lower content of ethyl acetate (Table 5).

When it comes to higher alcohols (Table 6), the beers produced by yeast rehydrated with PTWAir contained less of 2-methyl-1-propanol (*Sc* S33), 2-phenylethanol and 1-nonanol (*Sc* US-05). In turn, the beers obtained with yeast rehydrated with PTWN were characterized by reduction of 2,3-butanediol (*Sc* T58) and 2-phenylethanol concentrations (*Sc* US-05).

In the case of terpenes, it was observed that yeast rehydrated in the presence of PTWs caused changes in the amount of these aroma compounds in beer (Table 6). PTWAir treatment of yeast generally increased the presence of terpenes. These trends can be seen in the case of level of linalool in beers fermented with *Sc* US-05, and geraniol in samples fermented with *Sc* US-05 and *Sc* S33. PTWAir and PTWN contributed to increasing the β-farnesene concentration in beer fermented with lager yeast strain *Sp* W34/70. Application of PTWN resulted in beer with higher level of geraniol (*Sc* T58), however, the opposite phenomenon was observed for this compound in beerfermented with the *Sc* S33 yeast strain. Among the individual strains it was noted that the beers fermented with *Sc* US-05 had highest content of β-damascenone, on the other hand the beers fermented with *Sc* S33 yeast strain had highest concentration of β-myrcene. The highest amount of geraniol content was observed in beers fermented with *Sc* T58 yeast strain.

## 4. Discussion

The available literature data show that the use of dried yeast in brewing has several advantages and is gaining more and more popularity [7]. In addition, they also exhibit fermentation properties similar to their fresh counterparts [33]. Previous studies on the rehydration of dried yeast have focused on improving their quality during rehydration [11,12]. However, the available publications come from couple years ago or do not focus on the course of the fermentation process using these yeasts and the quality parameters of the finished beer.

In this paper, we presented how rehydrated yeast in the presence of PTWs influences the fermentation process and the quality of the finished beer. We showed that the rehydration of brewer’s yeast in the presence of various waters (PTWAir, PTWN, CW) significantly influenced the characteristics of yeast [15], contributed to changes during fermentation, and modified the chemical composition of the produced beers.

Rehydration of *Sp* W34/70 lager strain with PTWAir resulted in a better start of fermentation, compared to the samples fermented by yeast after PTWN or CW treatment. The beginning of fermentation process depends on many factors, mainly the yeast strain, its adaptability to the new environment, resistance to hop ingredients, the course of the entire process, etc. [34]. Studies by other scientists show that, a times, *Saccharomyces* yeast takes longer time to adapt to a new environment compared to other strains [35]. The obtained results presented in the article for the *Saccharomyces pastorianus* strain show that rehydration of yeast in the presence of PTWAir may contribute to better adaptation of yeast to the new environment and faster start of fermentation (Figure 1d). It can also be noticed that all of the yeast strains rehydrated with PTWN were characterized by a significantly slower fermentation rate, compared to control water. The greatest differences, in the case of rehydrated samples in the presence of PTWN, were observed for the *Sc* US-05 and *Sp* W34/70 yeasts strains.

The amount of ethanol produced is related to the use of sugar available in the wort by the yeast [36]. As fermentation progress, ethanol levels increase, and yeast cells are exposed to increasing levels of ethanol stress. Therefore, a great deal of research is carried out to maintain the proper physiological state of yeast during the entire fermentation process [37,38]. Rehydration of lager yeast with PTWN contributed to the production of beers with a lower alcohol content than in the other samples. Rehydration in this water did not contribute to the improvement of the quality of yeast cells. This is also in agreement with the obtained results of real extract, apparent extract, and ethanol yield parameters. The highest alcohol content was found in the beers after fermentation with *Sc* US-05 yeast strain, and this result is closest to the result obtained by Zdaniewicz et al. and Meier-Dornber et al. [35,39]. The lowest alcohol concentration was found in beers after fermentation with *Sc* S33 yeast strain. Statistically significant results were also observed for the amount of yeast biomass after the end of the fermentation process. If the yeast biomass is characterized by appropriate quality parameters, it can also be used for subsequent fermentation [40,41]. After rehydration, in the presence of PTWN, the yeast was characterized by a lower amount of biomass compared to the other samples.

The colour is an important quality attribute of beer as it is the first feature noticed by the consumer [42]. Barley malt has the greatest influence on colour, as this ingredient is used the most. Hops have little effect on the colour of beer [43]. The colour of a beer is influenced by many factors, such as boiling. During boiling, melanoidins are produced, the presence of which depends on the temperature, time, pH value of wort as well as the concentration of FAN and sugars [24]. Beers obtained after fermentation with yeast strains *Sc* US-05 and *Sc* S33 after rehydration in the presence of PTWAir, were characterized by significantly lower intensity of colour compared to other beer samples. An inverse relationship was observed in the case of lager beers where the colour in this case had the highest intensity. The beer samples after fermentation with *Sc* US-05 yeast strain had the lowest EBC units for colour, which is also related to the lowest FAN content. The colour of beer could have been influenced by the use of various yeast strain for fermentation. In studies conducted by Zdaniewicz et al. [35] it was observed that beers produced with the use of different yeast strain differed in colour. In, addition beers after fermentation with *Sc* US-05 strain were characterized by the lowest pH value compared to other strains. The obtained results for FAN and pH suggest that their dependence on the type of strain used for beer production. Plasma-treated water did not significantly change these parameters (FAN and pH).

Maltose is one of the key sugars during the fermentation of a brewing wort [44]. In turn, glucose and fructose are sugars that are very well fermented by yeast and used the fastest [45]. In samples of beers fermented with yeast after rehydration in the presence of PTWN, significantly higher levels of maltose were obtained compared to the other samples. Significantly lower maltose content was observed in samples with increased alcohol content. This trend also occurred in samples fermented with *Sp* W34/70 yeast strain rehydrated with PTWAir, these beers were characterized by an increased alcohol content, and therefore by a lower level of maltose. We also noted that the yeast after rehydration with PTWAir started to use the available maltose from the wort the most quickly. The beers obtained after fermentation with *Sc* US-05 yeast strain contained the lowest concentration of unfermented maltose (Table 4) and the highest of ethanol (Table 3). This observation of the results is reflected in the research of Gobbi et al. [46] where the analyzed *S. cerevisiae* strains showed high maltose fermentation capacity. The highest content of maltose was found in the *Sp* W34/70 samples. In the studies presented by Magalhaes et al. [47], a similar use of maltose was obtained in the case of the *S. pastorianus* strains.

Glycerol is not present in the wort but it is formed by microorganisms during fermentation (Table 4). The amount of produced glycerol depends on the yeast strains and fermentation temperature [48,49]. Glycerol contributes to the consistency, complexity, and fullness of the beverages. Additionally, it influences the intensity of the taste of the obtained beer [50]. The rehydration of yeast with PTWs made it possible to obtain beers containing different amounts of glycerol. After rehydration with PTWAir, a higher level of glycerol was observed in the beers fermented with the *Sc* S33 yeast strain, a similar trend was maintained for the samples with *Sp* W34/70 yeast strain. Rehydration with PTWN contributed to the reduction in glycerol content in beers produced with the use of the *Sc* US-05 yeast strain. In studies conducted by other scientists it was found that the increased aeration of the wort during fermentation also promotes the production of increased glycerol content [49]. Subjecting cells to stress factors may contribute to the overproduction of glycerol [51]. The obtained results of glycerol content are similar to those obtained by other scientists [52].

During the fermentation process, two main compounds are produced: ethanol and carbon dioxide. These products are above the odor thresholds for many beers and impart mouthfeel, body, and warmth to final product [2]. However, these are not the only fermentation products. A whole range of volatile compounds are formed, the profile of which is a function of both the yeast strain used and the composition of the finished wort [43]. These substances are present only in low concentrations; however, they are responsible for the aroma of beer [53]. Esters, higher alcohols, terpenes, and acids are the main compounds that influence the aroma of alcoholic beverages. The most important group of flavor components are esters from the fermentation process. They give beer fruity, floral, solvent flavors and aromas [3]. The flavor of beer is critical to its overall consumer acceptance. Therefore, the comparison of the flavor compounds in beer is difficult to understand from a sensory point of view without assessing the odor thresholds of each of the analytes [54]. The odor threshold values for the individual compounds are given in Table 4, 5, 6 and are in accordance with literature [2,32]. It was noted that ethyl acetate (sweet, solvent) has a high odor threshold, and the values presented in the article are below the detection threshold for this compound. A similar situation was observed for the remaining esters. As for higher alcohols, only the values for 2-methyl-1butanol (onion) are above the odor threshold for this compound in the obtained beers. Furthermore, the analysis of volatile compounds leads to the observation that the beers fermented using the *S. pastorianus* strain produced lower levels of esters and higher alcohols compared to the *S. cerevisiae* strain. In *S. pastorianus*—fermented beers, the content of ethyl acetate, 2-phenylethyl acetate, was significantly lower than in beers fermented with *Sc* US-05 yeast strain. Ravasio et al. [55] received a similar result in their research. Beers obtained with yeast strain *Sc* US-05 after rehydration with PTWAir were characterized by a lower content of 2-phenylethyl acetate and ethyl decanoate compared to beer obtained with this yeast strain after rehydration with control water (CW). A similar situation was observed in the case of higher alcohol, where samples with yeast strain *Sc* US-05 after rehydration with PTWAir had lower content of 2-phenylethanol and 1-nonanol. This could suggest that PTW alters cell’s metabolism. All flavor active ingredients in the beer must be properly controlled and kept within specified limits. Their relative concentration may impart favorable or undesirable flavor characteristics, and a slight alteration thereof may result in a completely different taste of the beer [56]. Therefore, it is very important to manage fermentation responsibly in order to maintain the high quality of the end product: beer. Fermentation is carried out so that the concentration of negative flavor component is lower than their threshold levels, as well as ensuring that the presence of appropriately selected amounts of higher alcohols and esters has a positive effect on the final product [57]. PTW water may affect metabolic processes in cells during rehydration thus influencing their formation during fermentation. It may be another method of modifying the beer volatile compounds profile (its quality), in addition to the currently used methods such as yeast cultures, varieties of hops or modifications of beer production technology.

Hops (*Humulus lupulus L.*) are essential in the brewing process to give the beer the right sensory properties [58]. The main ingredients of hops oil are terpenes, which account for up to 3% of the hops [59]. The composition of hop oil consists mainly of terpene hydrocarbons such as β-myrcene, α-humulene, β-caryophyllene and β-farnesene [59]. Depending on the style of beer, terpene alcohols are the dominant terpenes due to their hydrophilic properties, especially geraniol and linalool [60]. Interactions between hops and *Saccharomyces* yeast during fermentation may cause changes in the number of volatile compounds produced [61]. Studies by other scientists have shown that brewer’s yeast is characterized by low β-D-glucosidase activity during beer fermentation, but some strains exhibit exo-1,2-β-glucanase activity, which has the ability to hydrolyze glycosides. The beers produced with the use of *Sc* US-05 yeast strain after rehydration with PTWAir were characterized by a higher content of linalool and geraniol compared to control samples. Higher content of β-farnesene was also observed in the case of beer fermented with the lager yeast strain *Sp* W34/70 after rehydration with PTWAir and PTWN. This may indicate that the yeasts in the presence of these waters (PTW) during the fermentation have a greater possibility of interacting [2] with the hop compounds, which could have contributed to an increase in their content in the final product. Currently, for the purpose of this study, non-*Saccharomyces* yeasts with β-glucosidase activity are used, which when introduced into the hopped wort, release glycosidic precursors of aromas derived from hops [61].

## 5. Conclusions

The presented results describe the effect of brewing yeasts rehydration medium treatment on fermentation performance and beer quality. In the obtained results, it was observed that PTWAir significantly contributed to the earlier start of fermentation kinetics in the case of the *S. pastorianus* yeast strain. Additionally, yeast rehydrated in the presence of PTWAir contributed to the production of beers with higher quality parameters. We have noticed that plasma water treatment before of yeast rehydration can improve the volatile compounds formation during fermentation, especially terpenes. This was seen in the case of ale yeasts characterized by the activity of glycosidase, which plays a key role in the interacting with several component of the hop flavor during fermentation. This situation can be also used in the case of wine yeasts because they are characterized by a higher glycosidase activity. In the case of using PTWN, it was observed that it slightly contributed to the deterioration of the quality parameters of the obtained beers, which was already noticeable in the case of the quality parameters of yeast in our previous article.

## Figures and Tables

**Table 1:** The composition of wort before fermentation.

	Original Gravity [°P]	IBU	pH	Colour [EBC]	FAN [mg/L]	Ca^2+^ [mg/L]	Zn^2+^ [mg/L]	Mg^2+^ [mg/L]	Fructose [g/L]	Glucose [g/L]	Saccharose [g/L]	Maltose [g/L]	Citric Acid [g/L]	Malic Acid [g/L]	Succinic Acid [g/L]
	11.9 ± 0.09	26 ± 0.04	5.75 ± 0.02	14.3 ± 1.09	191 ± 31.36	21.6 ± 2.42	0.91 ± 0.17	93.7 ± 15.9	8.92 ± 1.17	7.74 ± 0.74	5.12 ± 0.46	37.1 ± 3.51	3.5 ± 2.48	2.7 ± 1.03	1.3 ± 0.21
Analytical Determinations		2.3.1.	2.3.4.	2.3.5.	2.3.6.	2.3.10.			2.3.9.				2.3.8.		

*n* = 3; International Bitterness Unit (IBU); free amino nitrogen (FAN).

**Table 2:** Quality parameters in beers fermented by *Saccharomyces cerevisiae* (T58, US-05, S33) and *Saccharomyces pastorianus* (W34/70) after rehydration with PTWAir, PTWN and control water (CW).

Parameters		Ethanol [% *v*/*v*]	Real Extract [% *w*/*w*]	Apparent Extract [% *w*/*w*]	Ethanol Yield [g/g]	Amount of Biomass [g of Dry Mass/dm]	Colour [EBC Units]	pH	FAN [mg/L]	Mg [mg/L]	Ca [mg/L]	Zn [mg/L]
*Saccharomyces cerevisiae* Safbrew US-05	PTWAir	5.33 ^a^ ± 0.02	4.12 ^ba^ ± 0.01	2.20 ^a^ ± 0.01	53.6 ^a^ ± 0.29	2.77 ^a^ ± 0.48	5.62 ^a^ ± 0.71	4.24 ^b^ ± 0.04	44.8 ^a^ ± 7.39	65.1 ^a^ ± 2.52	17.4 ^a^ ± 2.49	0.00 ^c^ ± 0.00
	PTWN	5.26 ^a^ ± 0.03	4.10 ^a^ ± 0.02	2.19 ^a^ ± 0.02	52.9 ^a^ ± 0.59	1.69 ^b^ ± 0.1	6.67 ^b^ ± 0.35	4.29 ^b^ ± 0.01	52.6 ^a^ ± 5.21	62.8 ^a^ ± 4.00	16.8 ^ab^ ± 1.72	0.14 ^a^ ± 0.19
	CW	5.35 ^a^ ± 0.05	4.17 ^b^ ± 0.03	2.24 ^a^ ± 0.01	54.2 ^a^ ± 0.81	2.75 ^a^ ± 0.68	6.81 ^b^ ± 0.08	4.26 ^b^ ± 0.02	51.9 ^a^ ± 6.64	71.8 ^b^ ± 2.39	22.4 ^a^ ± 2.03	0.27 ^a^ ± 0.08
*Saccharomyces cerevisiae* Safbrew T58	PTWAir	4.69 ^b^ ± 0.04	4.55 ^c^ ± 0.04	2.86 ^b^ ± 0.03	49.5 ^c^ ± 0.67	2.46 ^c^ ± 0.53	7.87 ^b^ ± 0.38	4.65 ^a^ ± 0.03	73.1 ^b^ ± 13.05	85.8 ^b^ ± 2.87	17.7 ^a^ ± 3.23	0.09 ^a^ ± 0.05
	PTWN	4.66 ^b^ ± 0.03	4.61 ^d^ ± 0.09	2.92 ^c^ ± 0.09	49.3 ^c^ ± 0.68	1.54 ^d^ ± 0.49	7.22 ^b^ ± 0.55	4.56 ^a^ ± 0.04	77.2 ^b^ ± 8.18	75.7 ^b^ ± 4.54	20.8 ^a^ ± 1.78	0.29 ^a^ ± 0.17
	CW	4.78 ^c^ ± 0.04	4.50 ^c^ ± 0.05	2.76 ^b^ ± 0.06	50.5 ^d^ ± 0.38	2.16 ^c^ ± 0.28	8.12 ^c^ ± 1.34	4.64 ^a^ ± 0.02	72.7 ^b^ ± 8.54	78.8 ^b^ ± 4.54	21.9 ^a^ ± 3.92	0.04 ^c^ ± 0.03
*Saccharomyces cerevisiae* Safbrew S33	PTWAir	4.38 ^f^ ± 0.01	5.32 ^e^ ± 0.02	3.75 ^d^ ± 0.02	51.8 ^f^ ± 0.62	2.38 ^a^ ± 0.27	8.57 ^c^ ± 0.22	4.59 ^a^ ± 0.06	61.4 ^a^ ± 6.96	75.0 ^b^ ± 1.85	16.9 ^ab^ ± 0.38	0.43 ^ab^ ± 0.02
	PTWN	4.30 ^f^ ± 0.01	5.31 ^e^ ± 0.02	3.73 ^d^ ± 0.04	51.8 ^f^ ± 0.14	1.15 ^b^ ± 0.30	8.95 ^c^ ± 0.06	4.59 ^a^ ± 0.01	66.2 ^a^ ± 4.83	80.4 ^b^ ± 4.87	20.6 ^a^ ± 1.1	0.18 ^ab^ ± 0.11
	CW	4.38 ^f^ ± 0.04	5.30 ^f^ ± 0.02	3.71 ^d^ ± 0.03	51.9 ^f^ ± 0.41	2.76 ^a^ ± 0.21	9.20 ^c^ ± 1.3	4.58 ^a^ ± 0.01	59.2 ^a^ ± 8.13	74.8 ^b^ ± 14.2	16.9 ^ab^ ± 1.59	0.36 ^ab^ ± 0.04
*Saccharomyces pastorianus* Saflager W34/70	PTWAir	4.79 ^c^ ± 0.06	4.38 ^c^ ± 0.11	2.67 ^b^ ± 0.15	49.6 ^c^ ± 1.67	1.95 ^e^ ± 0.19	7.95 ^b^ ± 1.24	4.63 ^a^ ± 0.08	136 ^c^ ± 7.99	80.5 ^c^ ± 9.33	21.7 ^a^ ± 2.24	0.64 ^b^ ± 0.05
	PTWN	4.51 ^b^ ± 0.08	4.66 ^d^ ± 0.13	3.00 ^c^ ± 0.16	48.8 ^e^ ± 0.15	0.76 ^f^ ± 0.06	6.32 ^a^ ± 0.2	4.56 ^a^ ± 0.01	155 ^c^ ± 12.9	72.9 ^bc^ ± 1.98	18.1 ^a^ ± 1.29	0.59 ^b^ ± 0.07
	CW	4.77 ^c^ ± 0.03	4.39 ^c^ ± 0.13	2.66 ^b^ ± 0.13	49.6 ^c^ ± 1.07	2.21 ^e^ ± 0.08	6.22 ^a^ ± 0.31	4.59 ^a^ ± 0.03	150 ^c^ ± 3.34	70.5 ^bc^ ± 3.32	17.6 ^a^ ± 0.67	0.47 ^b^ ± 0.02
Significance	Strain	***	***	***	***	***	***	***	*	***	ns	**
	Water	***	***	***	***	**	***	ns	ns	ns	ns	ns
	Strain × Water	***	***	***	***	***	***	*	**	***	**	**

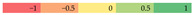
Values with different superscript roman letters (a–i) in the same column indicate statistically significant differences at *p* < 0.05; *n* = 4; ns—not significant; 0.001 ***; 0.01 **; 0.05 *. Color determination from lowest (0%) to highest (100%) concentration of parameters. The lowest concentration of a specific parameter in each column is in the darkest red and the highest content is in the darkest green.

**Table 3:** Concentration of sugars and organic acids in beers fermented by *Saccharomyces cerevisiae* (T58, US-05, S33) and *Saccharomyces pastorianus* (W34/70) after rehydration with PTWAir, PTWN and control water (CW).

Parameters		Maltose [g/L]	Fructose [g/L]	Glucose [g/L]	Saccharose [g/L]	Glycerol [g/L]	Acetic Acid [g/L]	Lacid Acid [g/L]	Malic Acid [g/L]	Citric Acid [g/L]	Succinic Acid [g/L]
*Saccharomyces cerevisiae* Safbrew US-05	PTWAir	0.51 ^a^ ± 0.36	6.54 ^c^ ± 0.15	1.94 ^acd^ ± 0.07	1.36 ^abe^ ± 0.03	5.49 ^a^ ± 1.01	0.08 ^a^ ± 0.01	0.03 ± 0.00	0.12 ^c^ ± 0.01	0.13 ± 0.01	0.03 ^a^ ± 0.00
	PTWN	1.78 ^c^ ± 0.21	4.81 ^b^ ± 0.07	2.73 ^be^ ± 0.21	1.31 ^abe^ ± 0.87	4.42 ^b^ ± 0.11	0.09 ^ab^ ± 0.003	0.02 ± 0.00	0.09 ^bc^ ± 0.00	0.02 ± 0.00	0.03 ^a^ ± 0.01
	CW	0.06 ^a^ ± 0.01	2.43 ^a^ ± 0.04	2.37 ^ab^ ± 0.44	0.15 ^c^ ± 0.13	5.55 ^a^ ± 0.43	0.07 ^ab^ ± 0.01	0.03 ± 0.00	0.09 ^b^ ± 0.00	0.02 ± 0.00	0.02 ^a^ ± 0.00
*Saccharomyces cerevisiae* Safbrew T58	PTWAir	0.07 ^a^ ± 0.02	6.91 ^c^ ± 0.85	3.69 ^f^ ± 0.05	1.07 ^ade^ ± 1.3	ND ± 0.00	0.11 ^ab^ ± 0.01	0.02 ± 0.00	0.01 ^a^ ± 0.00	0.05 ± 0.00	0.01 ^bc^ ± 0.01
	PTWN	0.06 ^a^ ± 0.01	8.74 ^d^ ± 0.45	2.59 ^abe^ ± 0.06	0.38 ^cd^ ± 0.23	ND ± 0.00	0.08 ^ab^ ± 0.01	0.07 ± 0.01	0.02 ^a^ ± 0.01	0.04 ± 0.00	0.03 ^a^ ± 0.01
	CW	1.62 ^c^ ± 0.39	6.81 ^c^ ± 0.34	3.10 ^ef^ ± 0.67	3.09 ^f^ ± 0.02	ND ± 0.00	0.11 ^ab^ ± 0.01	0.03 ± 0.00	0.02 ^a^ ± 0.00	0.31 ± 0.01	0.04 ^c^ ± 0.01
*Saccharomyces cerevisiae* Safbrew S33	PTWAir	0.26 ^ab^ ± 0.20	3.28 ^b^ ± 0.91	0.06 ^g^ ± 0.00	1.68 ^ab^ ± 0.01	6.68 ^c^ ± 1.06	0.08 ^ab^ ± 0.01	0.03 ± 0.00	0.03 ^a^ ± 0.01	0.04 ± 0.01	0.03 ^a^ ± 0.01
	PTWN	4.16 ^d^ ± 0.59	2.27 ^ab^ ± 1.4	1.29 ^c^ ± 0.08	0.49 ^cde^ ± 0.08	5.94 ^a^ ± 0.85	0.05 ^ab^ ± 0.01	0.03 ± 0.00	0.03 ^a^ ± 0.00	0.05 ± 0.00	0.04 ^c^ ± 0.00
	CW	6.81 ^e^ ± 0.58	0.33 ^a^ ± 0.01	1.95 ^acd^ ± 0.01	1.52 ^ab^ ± 0.08	6.11 ^a^ ± 0.52	0.09 ^ab^ ± 0.004	0.03 ± 0.00	0.03 ^a^ ± 0.01	0.05 ± 0.00	0.04 ^c^ ± 0.00
*Saccharomyces pastorianus* Saflager W34/70	PTWAir	4.42 ^d^ ± 0.44	0.39 ^a^ ± 0.02	2.21 ^abd^ ± 0.79	1.82 ^ab^ ± 0.61	8.93 ^d^ ± 0.64	0.12 ^b^ ± 0.005	0.02 ± 0.01	ND ± 0.00	0.02 ± 0.00	ND ± 0.00
	PTWN	8.55 ^f^ ± 1.31	ND ± 0.00	2.52 ^abe^ ± 0.19	2.07 ^b^ ± 0.14	8.46 ^e^ ± 0.74	0.11 ^b^ ± 0.01	0.02 ± 0.01	ND ± 0.00	0.03 ± 0.00	0.01 ^b^ ± 0.00
	CW	5.41 ^e^ ± 0.93	ND ± 0.00	1.49 ^cd^ ± 0.71	0.24 ^cd^ ± 0.02	8.03 ^e^ ± 0.55	0.11 ^b^ ± 0.01	0.19 ± 0.11	ND ± 0.00	0.03 ± 0.00	ND ± 0.00
Significance	Strain	***	***	**	**	***	*	ns	***	ns	***
	Water	***	***	ns	*	*	ns	ns	ns	ns	ns
	Strain × Water	****	***	*	***	***	*	ns	***	ns	***

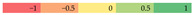
Values with different superscript roman letters (a–f) in the same column indicate statistically significant differences at *p* < 0.05; *n* = 4; ns—not significant; 0.001 ***; 0.01 **; 0.05 *. ND—Not detected. Color determination from lowest (0%) to highest (100%) concentration of parameters. The lowest concentration of a specific parameter in each column is in the darkest red and the highest content is in the darkest green.

**Table 4:** Esters in beers fermented by *Saccharomyces cerevisiae* (T58, US-05, S33) and *Saccharomyces pastorianus* (W34/70) after rehydration with PTWAir, PTWN and control water (CW).

Compound [ug/L]		Ethyl Acetate	Ethyl Hexanoate	Ethyl Octanoate	2-phenylethyl Acetate	Ethyl Decanoate	Ethyl Dodecanoate
LRI		614	986	1180	1228	1397	1581
*Saccharomyces cerevisiae* Safbrew US-05	PTWAir	1102 ^c^	3.37 ^a^	5.81 ^a^	378.2 ^c^	35.1 ^ab^	0.42 ^b^
	PTWN	775.7 ^b^	2.51 ^a^	5.89 ^a^	680.8 ^c^	35.1 ^ac^	1.15 ^a^
	CW	1140 ^c^	3.37 ^a^	5.89 ^a^	983.6 ^b^	41.7 ^c^	1.22 ^a^
*Saccharomyces cerevisiae* Safbrew T58	PTWAir	447.8 ^ab^	ND	5.74 ^a^	213.2 ^a^	10.5 ^c^	0.56 ^b^
	PTWN	372.7 ^ab^	ND	4.41 ^a^	257.4 ^a^	29.6 ^d^	1.29 ^ab^
	CW	427.7 ^ab^	ND	ND	169.5 ^d^	25.7 ^c^	1.17 ^ab^
*Saccharomyces cerevisiae* Safbrew S33	PTWAir	249.5 ^a^	ND	ND	685.5 ^c^	20.3 ^ab^	1.00 ^b^
	PTWN	720 ^abc^	ND	ND	641.8 ^c^	24.2 ^b^	1.29 ^ab^
	CW	685.2 ^abc^	ND	ND	912.2 ^b^	31.8 ^ab^	1.32 ^ab^
*Saccharomyces pastorianus* Saflager W34/70	PTWAir	407.7 ^ab^	ND	ND	125.6 ^a^	18.6 ^a^	1.33 ^a^
	PTWN	362 ^ab^	ND	ND	106.3 ^a^	10.1 ^ab^	1.31 ^a^
	CW	248.7 ^a^	ND	ND	164.3 ^d^	17.2 ^ab^	1.31 ^a^
OT		5000	6.4	15	3940	510	1000
Significance	Strain	**	**	***	**	*	*
	Water	ns	ns	**	**	ns	**
	Strain × Water	*	*	*	*	ns	*

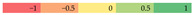
Values with different superscript roman letters (a–d) in the same column indicate statistically significant differences at *p* < 0.05; *n* = 4; ns—not significant; 0.001 ***; 0.01 **; 0.05 * ND—Not detected. LRI—linear retention index; the amount of components was determined. OT—Odour thresholds in beer [32]. Color determination from lowest (0%) to highest (100%) concentration of parameters. The lowest concentration of a specific parameter in each column is in the darkest red and the highest content is in the darkest green.

**Table 5:** Higher alcohol in beers fermented by *Saccharomyces cerevisiae* (T58, US-05, S33) and *Saccharomyces pastorianus* (W34/70) after rehydration with PTWAir, PTWN and control water (CW).

Compound [ug/L]		2-Methyl-1-propanol	2-Methyl-1-butanol	2,3-Butanediol	1-Hexanol	2-Phenylethanol	1-Nonanol
LRI		617	740	768	865	1084	1156
*Saccharomyces cerevisiae* Safbrew US-05	PTWAir	6.05 ^a^	2933 ^ac^	0.66 ^b^	17.4 ^a^	115 ^cd^	3.59 ^a^
	PTWN	12.4 ^bc^	2917 ^ac^	0.67 ^b^	22.4 ^ab^	153 ^d^	25.7 ^b^
	CW	14.8 ^bc^	3100 ^a^	0.67 ^b^	21.2 ^ab^	194 ^e^	25.6 ^b^
*Saccharomyces cerevisiae* Safbrew T58	PTWAir	ND	3049 ^ac^	0.51 ^bcd^	16.4 ^a^	57.5 ^a^	28.8 ^b^
	PTWN	ND	2917 ^ac^	0.17 ^acd^	15.7 ^a^	77.5 ^ab^	27.5 ^b^
	CW	ND	3481 ^a^	0.67 ^b^	14.9 ^a^	75.9 ^a^	32.9 ^b^
*Saccharomyces cerevisiae* Safbrew S33	PTWAir	6.49 ^a^	1765 ^cd^	0.33 ^abcd^	20.5 ^ab^	73.5 ^a^	8.41 ^e^
	PTWN	12.2 ^bc^	1047 ^d^	0.16 ^ac^	21.9 ^ab^	ND	9.91 ^d^
	CW	19.1 ^c^	1226 ^d^	ND	26.7 ^b^	ND	11.6 ^d^
*Saccharomyces pastorianus* Saflager W34/70	PTWAir	ND	400.2 ^b^	ND	21.8 ^ab^	83.1 ^ab^	3.82 ^f^
	PTWN	ND	346.8 ^b^	ND	22.3 ^ab^	82.4 ^ab^	3.30 ^a^
	CW	ND	930.9 ^b^	ND	17.9 ^a^	86.8 ^ab^	3.10 ^a^
OT		4000	400	31	100	-	50
Significance	Strain	**	***	**	**	***	***
	Water	*	ns	ns	ns	ns	**
	Strain × Water	**	**	**	*	***	***

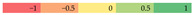
Values with different superscript roman letters (a–d) in the same column indicate statistically significant differences at *p* < 0.05; *n* = 4; ns—not significant; 0.001 ***; 0.01 **; 0.05 * ND- Not detected. LRI—linear retention index; the amount of components was determined. OT—Odour thresholds in beer [32]. Color determination from lowest (0%) to highest (100%) concentration of parameters. The lowest concentration of a specific parameter in each column is in the darkest red and the highest content is in the darkest green.

**Table 6:** Terpenes in beers fermented by *Saccharomyces cerevisiae* (T58, US-05, S33) and *Saccharomyces pastorianus* (W34/70) after rehydratation with PTWAir, PTWN and control water (CW).

Compound [ug/L]		β-Myrcene	Linalool	Geraniol	β-Damascenone	Humulene	β-Farnesene	Nerolidol
LRI		991	1092	1257	1384	1455	1462	1562
*Saccharomyces cerevisiae* Safbrew US-05	PTWAir	0.09 ^a^	0.15 ^b^	2.35 ^a^	0.31 ^b^	2.50	1.79 ^a^	0.68
	PTWN	0.09 ^a^	0.08 ^a^	0.32 ^b^	0.59 ^b^	2.91	0.89 ^b^	0.71
	CW	0.19 ^c^	0.07 ^a^	0.46 ^b^	0.14 ^b^	2.17	0.44 ^b^	0.73
*Saccharomyces cerevisiae* Safbrew T58	PTWAir	0.08 ^a^	0.1 ^a^	1.55 ^c^	ND	2.72	1.34 ^ab^	0.51
	PTWN	0.09 ^a^	0.18 ^b^	6.43 ^a^	ND	2.41	1.78 ^a^	0.69
	CW	0.08 ^a^	0.38 ^b^	0.94 ^b^	ND	2.41	1.8 ^a^	0.52
*Saccharomyces cerevisiae* Safbrew S33	PTWAir	0.11 ^b^	0.15 ^a^	5.72 ^a^	0.24 ^ab^	2.64	1.34 ^ab^	0.68
	PTWN	0.11 ^b^	0.03 ^a^	0.56 ^b^	0.08 ^a^	2.43	0.44 ^ab^	0.77
	CW	0.09 ^a^	0.03 ^a^	0.23 ^b^	0.09 ^a^	2.41	0.00 ^ab^	0.69
*Saccharomyces pastorianus* Saflager W34/70	PTWAir	0.08 ^a^	0.14 ^a^	1.27 ^a^	ND	2.25	1.79 ^a^	0.66
	PTWN	0.07 ^d^	0.12 ^a^	1.91 ^a^	ND	2.23	1.34 ^ab^	0.69
	CW	0.08 ^a^	0.08 ^a^	3.12 ^d^	ND	2.24	0.44 ^b^	0.69
OT		30	2.2	36	25	10	-	-
Significance	Strain	*	**	**	**	ns	*	ns
	Water	**	*	**	ns	ns	*	ns
	Strain × Water	*	*	**	*	ns	*	ns

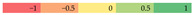
Values with different superscript roman letters (a–d) in the same column indicate statistically significant differences at *p* < 0.05; *n* = 4; ns—not significant; 0.001 ***; 0.01 **; 0.05 * ND—Not detected LRI—linear retention index; the amount of components was determined. OT—Odour thresholds in beer [2]. Color determination from lowest (0%) to highest (100%) concentration of parameters. The lowest concentration of a specific parameter in each row is in the darkest red and the highest content is in the darkest green.

## Data Availability

Data on the compounds are available from the authors.
